# Epidemiology, characteristics, and prognostic factors of lymphoplasmacyte-rich meningioma: a systematic literature review

**DOI:** 10.1186/s12885-023-11811-4

**Published:** 2024-01-22

**Authors:** Xiaoxi Zhu, Yanhua Liu, Weiwei Guo, Qi Liang, Chengliang Pan, Bin Tan, Ying Yu

**Affiliations:** 1 Department of Neurosurgery, First Hospital of Jilin University, 71 Xinmin Street, Changchun, 130021 Jilin China; 2 The Hospital of Northeast Normal University, Changchun, China

**Keywords:** Lymphoplasmacyte-rich meningioma, Epidemiology,Treatment, Recurrence, CNS disease

## Abstract

**Backgrounds:**

Lymphoplasmacyte-rich meningioma(LPM) is a rare subtype of meningioma with a low degree of malignancy and an overall preferable prognosis. The purpose of this article is to increase the understanding of the disease, reduce misdiagnosis, and improve prognosis.

**Methods:**

A search was conducted in the PubMed database for English articles published from 1993 to 2023. The keywords were "lymphoplasmacyte-rich (all fields) and meningioma (all fields) and English (lang)" and "lymphoplasmacyte-rich meningioma (title/abstract) and English (lang)".We further analyzed the clinical manifestations, imaging manifestations, pathological features, treatment strategies, and prognosis of LPM.The possible prognostic indicators were analyzed by the log-rank test and Pearson’s chi-squared test.

**Results:**

Fourteen reports with 95 LPM patients were included in this report, including 47 males and 48 females who were diagnosed between the ages of 9 and 79, with an average age of 45 years. The most common clinical manifestations are headache and limb movement disorders. In most cases, the tumor occurred on the convex portion of the brain. All tumors showed significant enhancement, with homogeneous enhancement being more common, and most patients showed peritumoral edema. Postoperative pathological EMA, LCA, and vimentin positivity were helpful for the final diagnosis of the patient. Log-rank tests showed a correlation between complete resection and better prognosis and recurrence.

**Conclusion:**

There is a lack of significant differences in the clinical symptoms and imaging manifestations of LPM compared to other diseases that need to be differentiated, and a clear diagnosis requires pathological examination. After standardized surgical treatment, the recurrence rate and mortality rate of LPM are both low. Complete surgical resection of tumors is associated with a better prognosis and lower recurrence rate.

## Introduction

Meningiomas are the most common primary tumor in the central nervous system (CNS), with a slow growth rate. In the latest 2021 World Health Organization (WHO) classification of central nervous system tumors, meningiomas are classified into 3 levels and a total of 15 subtypes, with grade 1 meningiomas being the most common in clinical practice [1]. The earliest report on LPM was in 1971 [2], and it was first included in the WHO classification in 1993. LPM belongs to a rare histological subtype of Grade 1 meningiomas, characterized by inflammatory cells with extensive infiltration and different proportions of meningioma cells, and infiltrating lymph plasma cells can even cover up the composition of meningeal epithelial cells [1]. The incidence rate of LPM is less than 1% [3] in meningiomas. Although LPM has been included in the WHO classification for 30 years, there are few cases report on LPM, with most of them being isolated cases report except for a few studies.The number of reported cases is too small to identify the clinical features that can help to definitively differentiate these meningiomas from typical meningiomas. The clinical, radiologic and pathologic features, and differential diagnosis of LPMs remain unclear.It is difficult to differentiate LPM from idiopathic hypertrophic pachymeningitis (IHP), inflammatory pseudotumor, Rosette-Doffmann disease and other diseases before surgery.Although it is clear that LPM is characterized by marked lymphoplasmacytic infiltration that often masks inconspicuous meningoepithelial components, its origin, whether neoplastic or inflammatory, remains controversial.In addition, factors associated with prognosis after surgical resection need to be further studied. In this article, we conducted a systematic review based on PubMed and analyzed the clinical manifestations, radiological manifestations, treatment strategies, pathological feature statistics and prognosis to help clinicians better understand this rare condition.Meanwhile, this paper discusses some characteristic manifestations of LPM such as edema and hematological abnormalities to have a positive effect on further study of this disease.

## Materials and methods

### Literature search

In this study, all patients were clearly diagnosed with LPM according to the WHO classification criteria for central nervous system tumors. We searched for articles related to LPM in the PubMed database. We reviewed the English literature published from 1993 to May 2023. The key words are "lymphoplasmacyte-rich (all fields) and meningioma (all fields) and English (lang)" and "lymphoplasmacyte-rich meningioma (title/abstract) and English (lang)". The inclusion criteria were as follows: (i) published in English, (ii) complete radiological examination results, (iii) confirmed as LPM through pathological examination, and (iv) treatment strategies, including subtotal resection and total resection.

### Article selection

**Fig. 1:**
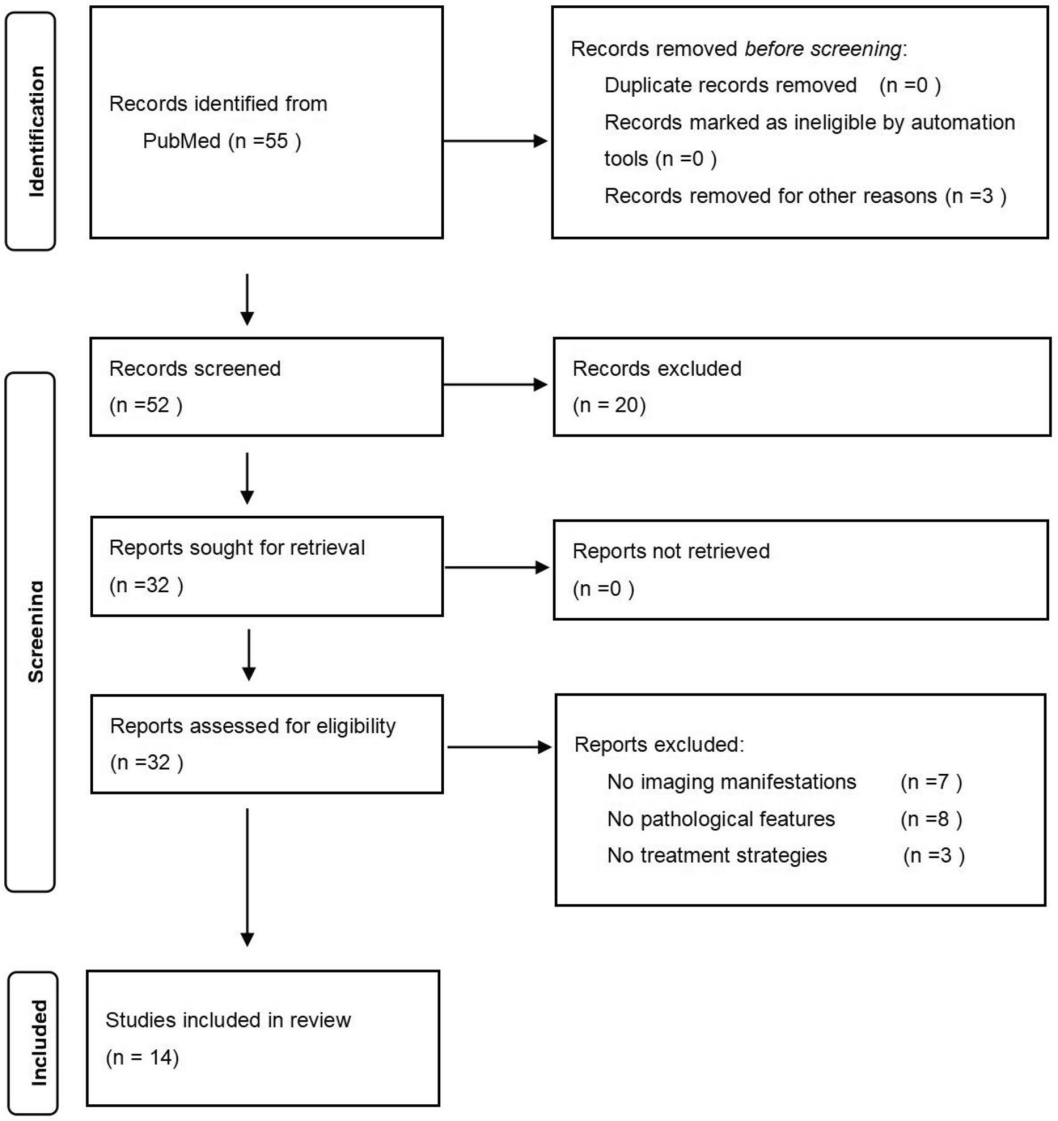
PRISMA flow diagram

A total of 55 related articles were retrieved, of which in 32 articles,patients were clearly diagnosed with LPM. According to our inclusion criteria, a total of 14 articles met our inclusion/exclusion criteria(Fig. 1). A total of 95 LPM patients were confirmed by postoperative pathological results, met all inclusion criteria and were included in the final analysis.To determine the validity of the selected articles, Zhu and Liu evaluated the data integrity of the case reports. If there were differences, the article was reviewed and discussed again until a consensus was reached. If the number of patients, diagnosis age, clinical symptoms, tumor location, imaging manifestations, treatment methods, and intervention types are mostly clearly described, the effectiveness of the case report is considered "good".

### Data statistics and analysis

We further analyzed its clinical manifestations, tumor location, radiological manifestations, pathological features, treatment strategies, and prognosis. The relapse-free survival (RFS) period was defined as the time from tumor resection to tumor relapse on imaging. Single-factor analysis using log-rank tests was used to evaluate intergroup differences and identify factors associated with recurrence in LPM patients. A *p* value of ≤ 0.05 was considered statistically significant.

## Results

### Epidemiological statistics

**Fig. 2:**
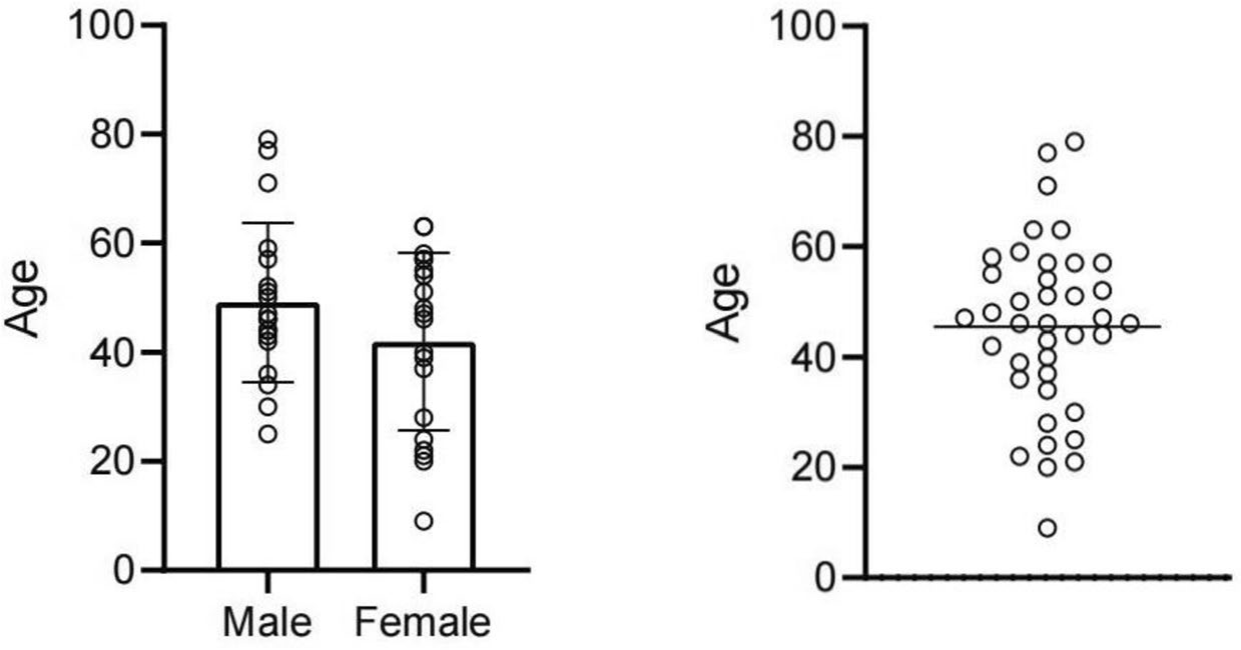
Age and sex distribution of lymphoplasmacyte-rich meningioma. Among these 39 LPM cases, the age of diagnosis ranged from 9 to 79 years (45.4 ± 15.7 years) and the male to female ratio was approximately 1 (19:20), mainly from 30 to 50 years old

In all 95 patients, the ratio of males to females was approximately 1 (47:48). The age at diagnosis was 9–79 years old, with an average age of 45 years. Most of the patients were diagnosed at 30–50 years old. In addition to the 56 patients with an unspecified age by Tao et al., the age at diagnosis in the remaining 39 patients was 45.4 ± 15.7 years old(Fig. 2).At the time of diagnosis, the most common symptoms of patients were headache, limb weakness, dizziness, blurred vision, numbness and seizures. Seven patients were diagnosed with a general medical examination. Tumors were mainly located in the convex part of the brain (48 cases) and lateral ventricles (6 cases). Twenty-eight patients had tumors located at the skull base, mainly distributed in the sphenoid ridge (9 cases), foramen magnum (6 cases), and tuberculum sellae (5 cases). A total of 34 patients had blood test results, of whom 9 patients showed hematological abnormalities and anemia. In T1-weighted imaging, 73 lesions were isointense or hypointense, and 1 lesion was hyperintense. On T2-weighted imaging, 9 lesions were hypointense, and 65 lesions were isointense or hyperintense. All 89 patients with enhanced MRI information showed enhancement, with homogeneous enhancement being the main manifestation (55 cases). Additionally, 19 patients showed heterogeneous enhancement, and in 15 patients, the enhancement status was not clearly indicated. Thirty-four patients underwent head CT examination, of whom 17 showed high-density lesions, 11 showed isodense lesions, and 6 showed mixed-density lesions. Tumor size was reported in a total of 63 patients, with 31 patients having a tumor length diameter less than 45 mm and 32 patients having a tumor length diameter greater than 45 mm. Fifty-one patients showed peritumoral edema (51/65), while 14 patients showed no significant peritumoral edema (14/65). A summary of the patient data is shown in Table 1,and Table 2 summarizes the demographics and clinical characteristics of these patients in detail.

**Table 1:** The summary of patient data

Variables	Number	%
Gender	95	
Male	47	49.5%
Fmale	48	50.5%
Age at diagnosis(years)	95	
< 45	47	49.5%
> = 45	48	50.5%
Mean	9–79	
Hematology	34	
Yes	9	26.5%
No	25	73.5%
Tumor size (mm)	63	
< 45	31	49.2%
> = 45	32	50.8%
T1-weighted	74	
Iso-/Hypointense	73	98.6%
Hyperintense	1	1.4%
T2-weighted	74	
Hypointense	9	12.2%
Iso-/Hyperintense	65	87.8%
Enhancement	89	
Homogeneous	55	61.8%
Heterogeneous	19	21.3%
Yes	15	16.9%
CT	34	
Hyperintense	17	50.0%
Isodense	11	32.4%
Mixed density	6	17.6%
Edema	83	
Yes	61	73.5%
No	22	26.5%
EMA	63	
+	62	98.4%
-	1	1.6%
PR	6	
+	3	50.0%
-	3	50.0%
CD3	20	
+	19	95.0%
-	1	5.0%
CD20	20	
+	19	95.0%
-	1	5.0%
CD138	7	
+	7	100.0%
-	0	0.0%
CD38	24	
+	23	95.8%
-	1	4.3%
LCA	46	
+	46	100.0%
-	0	0.0%
Vimentin	51	
+	50	98.0%
-	1	2.0%
S-100	14	
+	11	78.6%
-	3	27.3%
Ki67 + (%)	16	
< 5	10	62.5%
5–10	4	25.0%
> 10	1	6.3%
NA	1	6.3%
GFAP	13	
+	3	23.1%
-	10	76.9%
Surgery	95	
GTR	75	78.9%
STR	20	26.7%
Radiotherapy	80	
Yes	10	12.5%
No	70	87.5%
Recurrence	93	
Yes	7	7.5%
No	86	92.5%

*NA* Not available

**Table 2:** The detail demographic and clinical characteristics of these patients

Study	Year	Nb		Location	Age (years)	Gender	Cliencal symptoms	History (months)	Peripheral blood abnormalities	Maximum diameter (mm)	T1-weight	T2-weight	Enhancement	CT	Edema*
Wang,Y.B., et al. [25]	2018	1	1	Left frontal lobe	51	F	Left frontal scalp swelling	12	NA	35	Hypointense	Hyperintense	Yes	NA	NA
Yongjun, L., et al. [6]	2015	9	1	Right frontal lobe	39	F	Headache, dizziness, and vomiting(8/9)	NA	Anemia(4/9)	NA	Hypointense (9/9)	Hyperintense(7/9)	Yes(9/9)	Hyperdense(6/6)	Yes(5/6)
			2	Right lateral lobe	30	M	Epilepsy(3/9)					Hypointense (2/9)			
			3	Occipital sagittal sinus side	52	M	Vision loss(2/9)								
			4	Cerebellopontine angle	63	F	Hearing loss(1/9)								
			5	Cerebral falx	9	F									
			6	Sphenoid ridge	47	M									
			7	Right occiput	34	M									
			8	Left forehead	59	M									
			9	Left frontal temporalis lobe	54	F									
Tao, X., et al. [11] (multicenter study without individual information)	2017	56	56	Frontal convexity (19)	44.6 ± 12	F(28/56)	Headache or increased intracranial hypertension (24/56)	Average 2	NA	46 ± 16.7	Isointense (34/55)	Isointense (25/55)	Homogeneous enhancement (39/55)	Isodense (11/25)	- (13/56)
				Parietal convexity (10)		M(28/56)	Limb weakness (8/56)				Hypointense (21/55)	Hypointense (5/55)	Heterogeneous enhancement (16/55)	Hyperintense (10/25)	+ (21/56)
				Lateral ventricle (3)			Dizziness(7/56)					Hyperintense (25/55)		Mixed density (4/25)	+ + (8/56)
				Pineal region(2)			Blurred vision (7/56)								+ + + (14/56)
				Posterior fossa convexity (1)			Numbness (6/56)								
				Fourth ventricle(1)			Seizures (6/56)								
				Sphenoid ridge(6)			Memory loss (3/56)								
				Tuberculum sellae/Parasellar (5)			Diplopia(3/56)								
				Cerebellopontine angle (3)			Facial paralysis (3/56)								
				Petroclival region (3)			Hearing loss(2/56)								
				Foramen magnum (2)			Hyposmia (3/56)								
				Anterior skull base (1)			Asymptomatic(7/56)								
de Almeida, G.B. and G. Januário [26]	2022	1	1	Bilateral frontoparietal lobe	57	F	Left hemiparesis	NA	Normal	70	Isointense	Hypointense	Homogeneous enhancement	Hyperdense	Yes
Wang, H., et al. [7]	2021	1	1	Skull base to C6	44	M	Complaints of limb weakness	NA	Anemia	NA	Isointense	Isointense	Homogeneous enhancement	NA	NA
Gu, K.C., et al. [27]	2020	1	1	Right frontal lobe	36	M	Headache	NA	Normal	40	Hyperintense	Hypointense	Yes	Mixed density	Yes
Study	Year	Nb		Location	Age (years)	Gender	Cliencal symptoms	History (months)	Peripheral blood abnormalities	Maximum diameter (mm)	TIW1	T2W2	Enhancement	CT	Edema
Li, J., et al. [28]	2021	1	1	Skull base, tentorium, sella area, and C1-6	44	M	Extremities weakness	2	NA	NA	Isointense	Isointense	Homogeneous enhancement	NA	NA
Wang, Y.B., et al. [4]	2014	1	1	Ventricle	37	F	Generalized tonic–clonic seizure	5 Hours	NA	40	Hypointense	Hyperintense	Yes	NA	Yes
Kanno, H., et al. [15]	2011	1	1	Right jugular foramen	55	F	Hoarseness and dysphasia	NA	NA	15	Isointense	Hyperintense	Yes	Mixed density, homogeneous enhancement	NA
Yang, X., et al. [29]	2018	1	1	Lateral ventricle	47	F	Occipital-cervical region pain	6	NA	NA	Isointense	Hyperintense	Yes	NA	NA
Avninder, S., V. Gupta, and K.C. Sharma [30]	2008	1	1	The medulla and upper cervical cord mass	22	F	Headache	6	NA	35	Isointense	NA	Homogeneous enhancement	NA	NA
Majumdar, K., et al. [31]	2013	1	1	Right sphenoid rigid	50	M	Blurred vision	NA	Normal	50	NA	Hyperintense	Yes	NA	NA
Zhu, H.D., et al. [8]	2013	19	1	Left occipital lobe	20	F	NA	NA	Normal		NA	NA	Homogeneous enhancement (11/14)		+
			2	Right frontoparietal lobe	28	F			Hyperglobulinemia/Anemia				Heterogeneous enhancement (3/14)		+ + +
			3	Left temporal lobe	42	M			Normal						-
			4	Left frontotemporal lobe	48	F			Normal						NA
			5	Right frontal falx	77	M			Normal						-
			6	Right frontal lobe	46	M			Normal						+
			7	Left occipital lobe	46	M			Normal						-
			8	Right frontoparietal lobe	57	M			Normal						+
			9	Left sphenoid ridge	58	F			Normal						+ +
			10	Right frontal falx	79	M			Normal						-
			11	Right frontal falx	63	F			Normal						+
			12	Right frontal falx	71	M			Normal						+ +
			13	Right frontal lobe	46	F			Normal						+ +
			14	C1-2	25	M			Anemia						-
			15	Foramen magnum to C1-4	57	F			Normal						-
			16	Left cerebellum &tentorium	21	F			Anemia						-
			17	Right frontoparietal lobe	40	F			Normal						+
			18	C3-4	51	M			Normal						-
			19	Left frontoparietal lobe	24	F			Normal						+ + +
Fukui, K., et al. [32]	2021	1	1	C2-3	43	M	Neck pain	12	Hyperglobulinemia/Anemia		Isointense	Hyperintense	Homogeneous enhancement		NA

^*^-, no edema; + , equal to or smaller than the tumor size; +  + , surpassing the tumor size; +  +  + , nearly hemispheric edema

### Pathological characteristics and treatment methods

Immunohistochemical examination showed that most patients (62/63) were positive for epithelial membrane antigen (EMA), and 50/51 patients were positive for Vimentin. A total of 3/6 patients were positive for progesterone receptor(PR), 19/20 patients were positive for CD3, 19/20 patients were positive for CD20, 23/24 patients were positive for CD38, 7/7 patients were positive for CD138, 46/46 patients were positive for leukocyte common antigen (LCA), 11/14 patients were positive for S-100 protein, and 3/12 patients were positive for glial fibrillary acidic protein (GFAP). The vast majority of the patients had a Ki-67 labeling index less than 10% (14/16), with 10 cases being less than 5% and 4 cases being 5%-10%. The index size was not clearly indicated in one Ki67-positive patient. All patients underwent surgical treatment, with 2 patients receiving surgical treatment again after recurrence. Twenty patients underwent subtotal resection(STR) (20/95), and 75 patients underwent gross total resection(GTR) (75/95). Table 3 summarizes the pathological characteristics and treatment methods of these patients.

**Table 3:** The pathological features, therapy and follow-up of these patients

Study	Year	Nb		Pathology											Surgery	Following-up time(months)	Recurrence	0utcome	Radiotherapy
				EMA	CD3	CD20	CD138	CD38	LCA	Vimentin	S100	Ki67 + (%)	GFAP	PR					
Wang,Y.B., et al. [25]	2018	1	1	+	+	+	+					3		-	GTR	24	No	ANT	NA
Yongjun, L., et al. [6]	2015	9	1	+ (9/9)				+ (9/9)	+ (9/9)	+ (9/9)					GTR	42	No	ANT	No(9/9)
			2												GTR	34	No	ANT	
			3												STR	56	Yes(1 year)	ANT	
			4												GTR	23	No	ANT	
			5												GTR	34	No	ANT	
			6												STR	38	No	AWT	
			7												GTR	52	No	ANT	
			8												GTR	22	No	ANT	
			9												GTR	31	No	ANT	
Tao, X., et al. [11](multicenter study without individual information)	2017	56	56	+ (23/24)	+ (13/14)	+ (12/12)		+ (13/14)	+ (12/12)	+ (18/19)	+ (10/12)	1–5(5/9)	3		GTR (45/56)	5–97 (average41.5)	3/56(at 36 months)	Died (1/56)	Yes(6/56)
				-(1/24)	-(1/14)			-(1/14)		-(1/19)	-(2/12)	5–10(3/9)	5		STR (11/56)		5/56(at 60 months)	ANT at 36 months (52/56)	No(50/56)
												> 10(1/9)						ANT at 60 months (50/56)	
de Almeida, G.B. and G. Januário [26]	2022	1	1	+	+	+									GTR	12	No	ANT	NA
Wang, H., et al. [7]	2021	1	1	+	+	+	+		+			< 1	-	+	STR			Dead 2 weeks after operation for pneumonia	No
Gu, K.C., et al. [27]	2020	1	1	+	+	+	+	+	+	+	-	< 5	-	-	GTR	36	No	ANT	No
Li, J., et al. [28]	2021	1	1	+	+	+	+		+				-	+	STR	3	No	AWT	No
Wang, Y.B., et al. [4]	2014	1	1	+	+	+	+			+		+	-	-	GTR	3	No	ANT	NA
Kanno, H., et al. [15]	2011	1	1	+			+		+					+	GTR	9	No	ANT	NA
Yang, X., et al. [29]	2018	1	1	+			+								STR	3	No	AWT	Yes
Avninder, S., V. Gupta, and K.C. Sharma [30]	2008	1	1	+					+						GTR	12	No	ANT	NA
Majumdar, K., et al. [31]	2013	1	1	+		+			+	+	+				GTR	9	No	ANT	NA
Zhu, H.D., et al. [8]	2013	19	1	+ (19/19)					+ (19/19)	+ (19/19)			-(most)		GTR	Lost follow-up			Yes(3/19)
			2												GTR	63	No	ANT	No(16/19)
			3												STR	59	No	AWT	
			4												GTR	Lost follow-up			
			5												GTR	50	No	ANT	
			6												GTR	50	No	ANT	
			7												GTR	50	No	ANT	
			8												GTR	49	No	ANT	
			9												STR	46	No	AWT	
			10												GTR	45	No	ANT	
			11												GTR	41	No	ANT	
			12												GTR	39	No	ANT	
			13												GTR	39	No	ANT	
			14												GTR	37	No	ANT	
			15												STR	33	No	AWT	
			16												GTR	32	No	ANT	
			17												GTR	31	No	ANT	
			18												GTR	28	No	ANT	
			19												GTR	24	No	ANT	
Fukui, K., et al. [32]	2021	1	1	+						+		5	-		STR	12	Yes(at 12 months)	AWT	NA

### Follow-up, prognosis, and data analysis

**Fig. 3:**
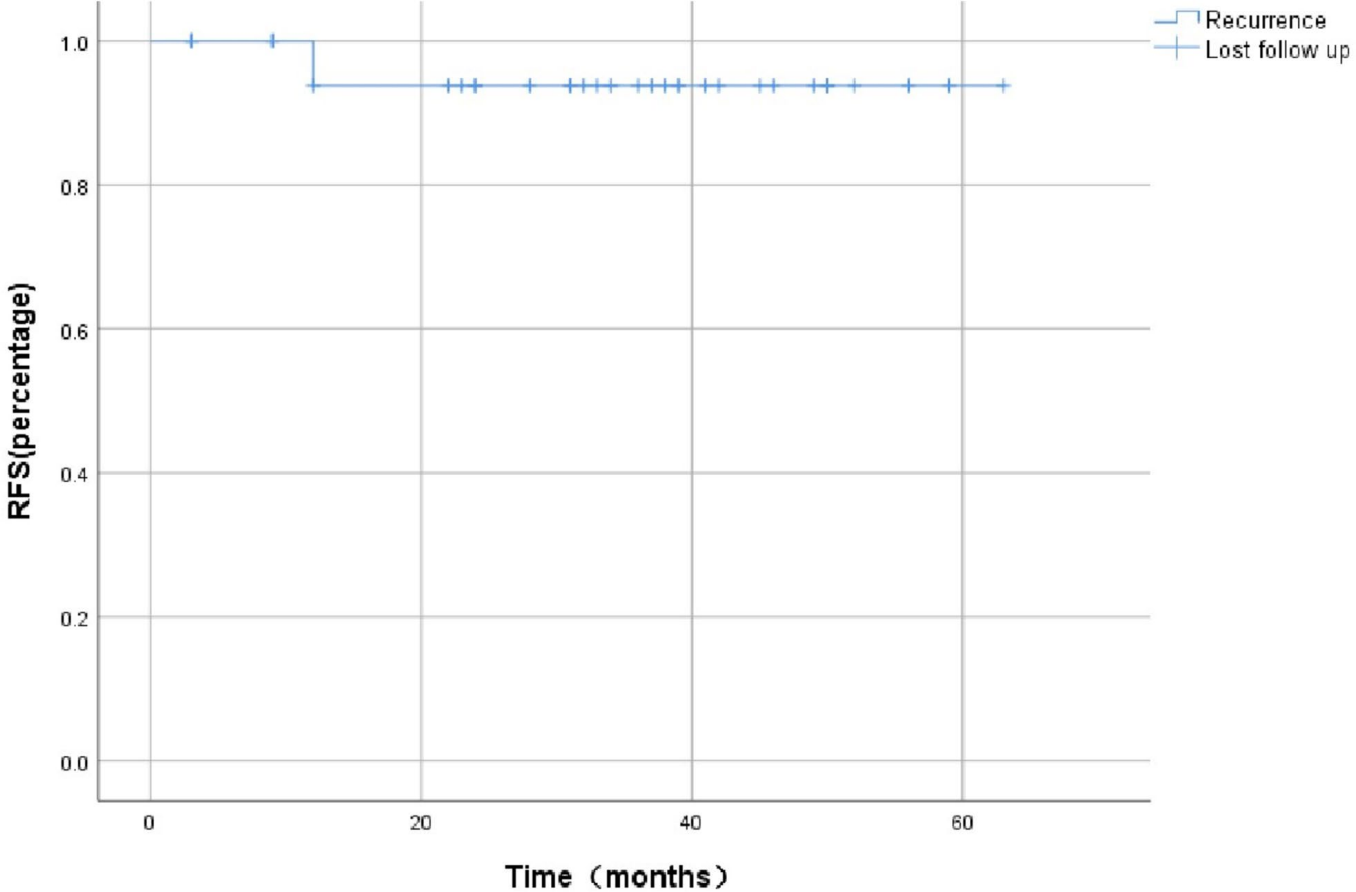
The Kaplan–Meier curves of RFS

The follow-up period was 3–97 months. During the follow-up period, 6 patients experienced local recurrence (6/94), and 1 patient died during the study period. Except for one patient who died of pneumonia two weeks after surgery and 2 patients lost to follow-up, the 3-year recurrence rate and 5-year recurrence rate were 5.4% (5/92) and 7.6% (7/92), respectively.A Kaplan–Meier curve was created to show the recurrence rate (Fig. 3). Two patients underwent surgical treatment again after recurrence but did not experience recurrence after the second surgery. Only one patient died of tumor recurrence within 5 years. The follow-up and prognosis of these patients are shown in Table 3. Single-factor analysis shows that peripheral blood abnormalities and whether they are completely removed are related factors that affect the improvement of postoperative symptoms in patients. Tumors that are completely removed have a relatively better prognosis. The surgical strategy also affects postoperative recurrence in patients, and patients who have undergone complete resection are less likely to experience recurrence after surgery.The results obtained from the log-rank test are presented in Table 4. The main factors affecting the surgical strategies are the tumor size and the site of tumor. Complete resection is most likely to be achieved in tumors with a size less than 45 mm and a growth site located outside the skull base.The results obtained from the Pearson’s chi-squared test are presented in Table 5. A total of 80 patients underwent postoperative radiation therapy, of whom 10 received postoperative radiation therapy. The main reason for receiving radiation therapy was that the surgery did not achieve complete resection. Although there is a report that tumors that have not been completely removed decrease in size after postoperative radiotherapy [4], statistical analysis suggests that postoperative radiotherapy has no significant correlation with prognosis or recurrence.

**Table 4:** The results of the log-rank test

Variable	*p*-value
Age(< 45)(*n* = 36)	
< 45	0.814
≥ 45	
Gender(*n* = 36)	
Male	0.164
Fmale	
Extent of resection(*n* = 36)	
GTR	0.003
STR	
Location(skull base or not)(*n* = 36)	
No skull base	0.528
Skull base	
Peripheral blood abnormalities(*n* = 21)	
Normal	0.046
Abnormal

**Table 5:** The results of the Pearson’s chi-squared test

Variable	GTR	STR	*p*-value
Age(*n* = 75)			
< 45	31	6	0.286
≥ 45	28	10	
Tumor size (mm)(*n* = 63)			
< 45	29	2	0.023
≥ 45	23	9	
Location(skull base or not)(*n* = 75)			
No skull base	43	6	0.008
Skull base	16	10	
Peritumoral edema(*n* = 58)			
Yes	30	6	0.568
No	17	5	
T2-weighted imaging signals(*n* = 64)			
Iso-/Hyperintense	45	12	0.650
Hypointense	5	2	

## Discussion

Meningiomas are the most common benign tumor in the brain, originating from arachnoid cap cells covering the brain and spinal cord. LPM is a rare WHO I meningioma subtype with a low prevalence of meningeal epithelial tumors and a high infiltration of inflammatory cells. The majority of patients with this type of meningioma are young to middle-aged. In our study, the patients' onset ages ranged from 9–79 years old, and there was no discernible sex difference. The number of males and females was basically the same (47 vs. 48), which is consistent with previous research results and shows differences from other types of meningiomas.The incidence rates for meningiomas globally seem to be more than twice as higher in women than in men. This type of tumor mainly occurs in the convex surface and can also be seen sporadically in the spinal canal and ventricles. It is commonly solitary, but there are also individual cases of sporadic or diffuse lesions. More than 1/4 of the patients with clearly recorded hematology examination results showed hematology abnormalities, which is where LPM is significantly different from other types of meningiomas. More than 70% of patients exhibit peritumoral brain edema, and approximately one-third of patients exhibit moderate to severe edema. Although only approximately 80% of patients have GTR and the vast majority of patients have not received postoperative radiotherapy or chemotherapy, the recurrence and mortality rates of this type of tumor are extremely low.

Depending on the tumor's location and biological function, LPM patients exhibit a variety of clinical manifestations. Headaches are the most typical clinical symptom of LPM sufferers. Other commom clinical symptoms include limb weakness, seizure, dizziness, blurred vision, etc. Additionally, in a small number of patients, the tumors were found on their general medical examination without overt clinical symptoms. The natural history of LPM usually exceeds 3 months, and a few patients may have a sudden onset due to the location of the lesion or infiltration and edema of inflammatory cells. Wang et al. reported a patient with a 5-h generalized tetanic spasm as the main manifestation [5]. The differences in these symptoms may be mainly related to the location of the tumor and the compression of brain tissue by the tumor itself and surrounding edema [6].

In our study, 78.5% (51/65) of patients showed the phenomenon of peritumoral edema. Tao et al. detailed peritumoral edema in their study, of which 76.8% (43/56) of patients showed significant peritumoral edema, and among the patients with peritumoral edema, more than half of the patients showed peritumoral edema exceeding the tumor size, and even 1/3 of the patients exhibited edema nearly hemispheric [4].In our studies, we were unable to accurately assess the volume of peritumoral edema or its relationship to tumor volume, as many case reports only provided a textual description or partial imaging data on the presence or absence of edema. The occurrence of edema in meningioma is not rare, and studies have shown that peritumoral cerebral edema occurs in approximately 37% to 68% of patients with intracranial meningioma other than the suprasellar area [33]. There are multiple explanations for the mechanism of this edema. First, due to the lack of tumoral blood supply, meningiomas secrete angiogenic factors (such as VEGF-A, endothelin-1, and caveolin-1), which can lead to increased permeability of tumoral vessels and development of the peritumoral pial vascular network, ultimately leading to alterations in the extracellular matrix and plasma protein leakage [34–38]. Second, large meningioma leads to brain compression, which leads to brain tissue ischemia and cytotoxic edema [33, 39]. However, the theory has obvious flaws, sometimes very small meningiomas cause extensive peritumoral edema. Other mechanisms of peritumoral edema include tumoral obstruction of veins [40] and sinuses and specific histological types of meningioma that produce eosinophilic and PAS positive inclusions and induce peritumoral edema through the osmotic mechanism [41, 42]. Osawa et al. classified meningothelial, transitional, and fibrous meningiomas as ‘WHO grade I common type’ and the other subtypes of grade I as ‘WHO grade I uncommon type’ [43]. They reported that the uncommon type had higher edema indices than the common type (69% vs. 34%). Recently, Park et al. found that IL-6 protein localized in the cytoplasm of the tumor cells, and was detected in 75% of edematous meningiomas, indicating that IL-6 expression may contribute to the formation of brain edema in meningiomas [44]. We hypothesize that massive infiltration of lymphocytes and plasma cells may play a central role in the development of the cerebral edema associated with LPM. Extensive edema may also hurt outcomes, although this was not validated in the analysis in this article. Edema brain tissue is more fragile than normal tissue, which increases the difficulty for surgeons to perform surgical procedures and further leads to the possibility of tiny residues that can serve as a basis for tumor recurrence; At the same time, the increased blood supply provided by the hyperplasia of vascular tissue in edematous brain tissue will also provide some aspect of the promotion of postoperative recurrence [45].

According to the literature, LPM is usually accompanied by abnormal peripheral blood. This special phenomenon has only been reported in two meningioma subtypes, LPM (WHO grade I) and choroid-like meningioma (WHO grade II) [2]. Abnormalities in the peripheral blood in LPM patients usually manifest as hyperglobulinemia and/or small cell hypochromic anemia [7, 8], which can usually return to normal after tumor resection.Hematologic abnormalities are not significantly related to the treatment and prognosis of LPM. Horten et al. [46]examined 5 cases considered meningiomas with extensive plasma cell-lymphocytic infiltrates. However, in the first of the 5 cases and another study by Gi et al. [47], only hypergammaglobulinemia with increased IgG was detected. The hypergammaglobulinemia is noteworthy, as it may reflect the possibility of localized neoplastic or nonneoplastic plasma cell dyscrasia or an immunoproliferative disorder at the meningeal site. Weidenheimet et al. [48] hypothesized that lymphoplasmacytic infiltration is an immune response to tumor antigenicity, while Gregorios et al. [49] hypothesized that these cells differentiate from totipotent mesenchymal cell clusters, as is the case with chronic and ongoing inflammation surrounding neoplastic meningeal epithelial components.Although hematologic bnormalities were identified in this study, the discussion of hematologic abnormalities can deepen our understanding of the pathogenesis of LPM. Kepes et al. [50] reported that choroid meningiomas are more likely to be accompanied by LPM and causes Castleman’s syndrome in children and young adults (delayed somatic and sexual development, hepatosplenomegaly, iron refractory hypochromic microcystic anemia, and bone marrow plasmacytosis with dysgammaglobulinemia). Thus we speculate that the particular histological manifestation and high proportion of dysgammaglobulinemia and/or iron refractory hypochromic microcysticanemia in LPM patients may imply similar pathogenesis of these two variants.In our study, there were 34 patients with clearly recorded haematology examination results, and more than 1/4 of the patients showed hematology abnormalities. This peripheral blood abnormalities may be secondary to the unusual immune response of the disease [9]. In this article,hematologic abnormalities may also mean a worse prognosis. It has been more than 40 years since LPM was included in the WHO classification, but the mechanism of massive infiltration of lymphocytes, plasma cells, and macrophages in this meningioma tissue remains unclear. More clinical case collection, analysis, and research may be able to provide a clear cause of this phenomenon, further suggest the origin of this type of meningioma, and provide guidance for its diagnosis and treatment.

The typical manifestations of LPM on MRI are hypointensity on T1-weighted images and hyperintensity on T2-weighted images. After enhancement, it shows homogeneous enhancement, often with obvious peritumoral brain edema and dural tail signals. The results are basically consistent with previous studie, while approximately 12.1% (9/74) of patients still exhibited hypointense T2-weighted images. All patients showed significant enhancement, but a considerable number of patients exhibited heterogeneous enhancement (19/89). The CT examination results of the patients did not show significant specificity, with half of the patients (17/34) exhibiting high-density shadows and approximately one-third of the patients still exhibiting isodense shadows. The cystic changes and heterogeneous enhancement in MRI manifestations increase the difficulty of preoperative diagnosis. Image enhancement is a helpful step to better identify the lesional tissues with lesions. Both of the tissue structures and pathological changes should be more visible after enhancement. Thus, the illuminance improvement for such images should be accounted for as well [51, 52].It would be of great interest to obtain the more typical imaging features of the LPM through the postprocessing of the images, which will help to guide diagnosis and preoperative planning.Studies have shown that in cardiovascular and retinal imaging, nonuniform contrast showed some effect [51, 53].Research has shown that most meningiomas with slow growth rates, especially those classified as WHO grade I, exhibit only moderate increases in glucose metabolism, making the detection results of ^18^F-flurodeoxyglucose(^18^F-FDG) PET unreliable [10]. Meningiomas often take up ^18^F-FDG at a similar or slightly lower rate than healthy grey matter. However, there has been a report of a case where the ^18^F-FDG uptake ratio (tumor/contralateral cortex) of the LPM was relatively high [11], at 1.38. The uptake of ^18^F-FDG may also increase due to inflammation, infection or granuloma. This feature is consistent with the inflammatory characteristics of LPM, but its clinical value needs further evaluation.

Pathological evaluation is the gold standard. It has been reported that EMA and vimentin staining can help to indicate the origin of the meningeal epithelium [7, 12] of tumors and distinguish LPM from other intracranial lesions, such as IHP, choroid meningioma, inflammatory pseudotumor and sinus histiocytosis with massive lymphadenopathy (SHML). In our study, tumor cells showed strong immunoreactivity to EMA and vimentin in the inflammatory background and diffuse infiltration of plasma cells and lymphocytes, which are typical LPM manifestations.

Considering that many intracranial masses may show characteristics similar to LPM, differential diagnoses should be considered, including IHP, choroid meningitis, inflammatory pseudotumor, and SHML. The pathological manifestations of IHP frequently include fibrosis and thickening of the dura mater, obvious infiltration of lymphocytes and plasma cells, and occasionally meningeal epithelial hyperplasia, which are similar to those of LPM [13–15]. Because IHP typically exhibits diffuse layered thickening or patchy features, the presence of local nodular changes can usually rule out this diagnosis [16]. Choroid meningiomas usually contain areas that are histologically similar to chordomas, eosinophils with cords or trabeculae, and a rich myxoid matrix background. Histological examination can assist in differential diagnosis [17]. Inflammatory pseudotumors should also be considered an important differential diagnosis because their clinical and imaging characteristics are similar to those of LPM. Inflammatory pseudotumor is a unique solid disease that is common in the lung or other organs. It is characterized by infiltration of different numbers of muscle fibroblast spindle cells and lymphoid plasma cells [18–20]. Among the reported primary intracranial inflammatory pseudotumors, meningeal lesions are the most common, even though their overall incidence rate is still low, making them rare [21–23]. When plasma cell granuloma forms a meningeal mass, it is possible to make a clear histological differentiation from LPM only when there is no meningeal epithelial component in the former [24]. The typical histopathological features of SHML are an obviously dilated sinus node, fully mature histiocytes, lymphocyte phagocytosis and plasma cell proliferation. Lymphoid plasma cell-rich meningiomas in the meninges and SHML have similar radiological features and pathological manifestations [25].

The follow-up data of patients show that the recurrence and mortality rates of LPM are very low, and patients are expected to achieve long-term survival after surgery. In our study, factors related to postoperative symptom improvement in patients mainly included whether the tumor was completely removed and whether the patient had peripheral blood abnormalities. Patients with GTR and those without peripheral blood abnormalities are more likely to achieve symptom relief. Further analysis of patient data suggests that the size of the tumor, as well as whether the tumor is located at the skull base, is closely related to the surgical treatment. Patients with a tumor length < 45 mm and nonskull base locations are more likely to achieve GTR.

There are still many difficulties in achieving a preoperative diagnosis of LPM through imaging, and an accurate diagnosis of LPM requires a comprehensive analysis of imaging and pathological results. Surgical resection is still the main treatment method, and the clinical outcomes of most patients are relatively good. For patients suspected of LPM, complete tumor resection should be achieved as much as possible while ensuring patient safety. There is no clear evidence to support the positive significance of postoperative radiotherapy and chemotherapy for the prognosis of the disease. Due to the pathogenesis of the disease, hormone or immunosuppressive drug treatment may be helpful, but further observation is still needed.

## Conclusion

LPM is a rare subtype of WHO grade I meningioma with low malignancy. After standardized surgical treatment, the recurrence rate and mortality rate are both low. It is difficult to make an accurate diagnosis before surgery solely based on clinical manifestations and imaging manifestations. Diagnosis requires surgical resection of the patient's tumor and completion of a pathological examination. According to our existing data, complete surgical resection of tumors is associated with a better prognosis and a lower recurrence rate.In tumors with smaller volumes and those located in nonskull bases, complete resection is more easily achieved. However, due to the small study cohort, the above conclusions are not entirely reliable. We should be aware that further larger cohort studies are needed to explore prognosis and recurrence.

## Data Availability

All data generated or analysed during this study are included in this published article.
